# Supplementary material for: POLB Regulates Proliferation and Apoptosis of Bovine Primary Myocytes
Source: Animals (Basel). 2024 Apr 28;14(9):1323. doi: 10.3390/ani14091323 (PMC11083859; doi:10.3390/ani14091323)
Supplement: Supplementary file 1 [file animals-14-01323-s001.zip › animals-2886102-supplementary.pdf]

Supplementary Materials for

POLB regulates proliferation and apoptosis of bovine primary myocytes

Geyang Zhang[1,2,†], Jiamei Wang[3,†], Yulong Li[1], Zijing Zhang[1], Xiangnan Wang[1], Fuying Chen[1], Qiaoting Shi[1], Yongzhen Huang[3], Eryao Wang[1,*] and Shijie Lyu[1,4,*]

Affiliations:

1 Institute of Animal Husbandry and Veterinary Science, Henan Academy of Agricultural Sciences, Zhengzhou, Henan 450002, China

2 College of Animal Science and Veterinary Medicine, Henan Agricultural University, Zhengzhou, Henan 450046, China

3 College of Animal Science and Technology, Northwest A&F University, Yangling 712100, China

4 The Shennong Laboratory, Zhengzhou, Henan 450002, China

*Correspondence: Shijie Lyu: sjlyu@outlook.com; Eryao Wang: eryaowang@outlook.com

† These authors contributed equally to the work.

This PDF file includes:

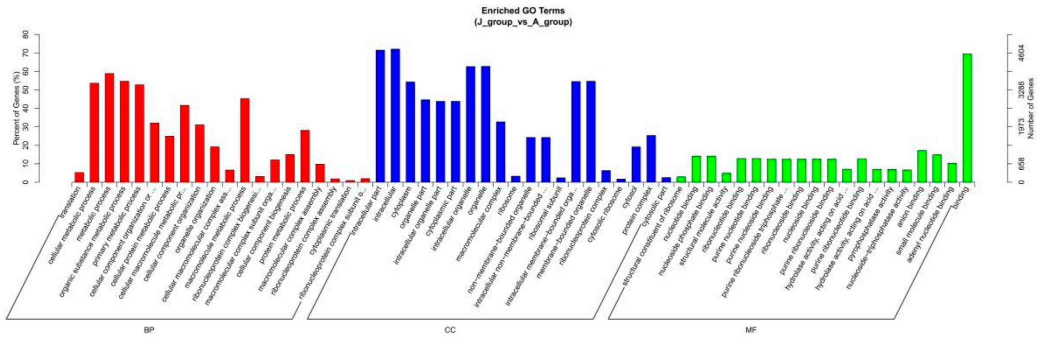

Figure S1.GO enrichment results

Note: BP represents biological processes, CC represents cellular components, and MF represents molecular functions.

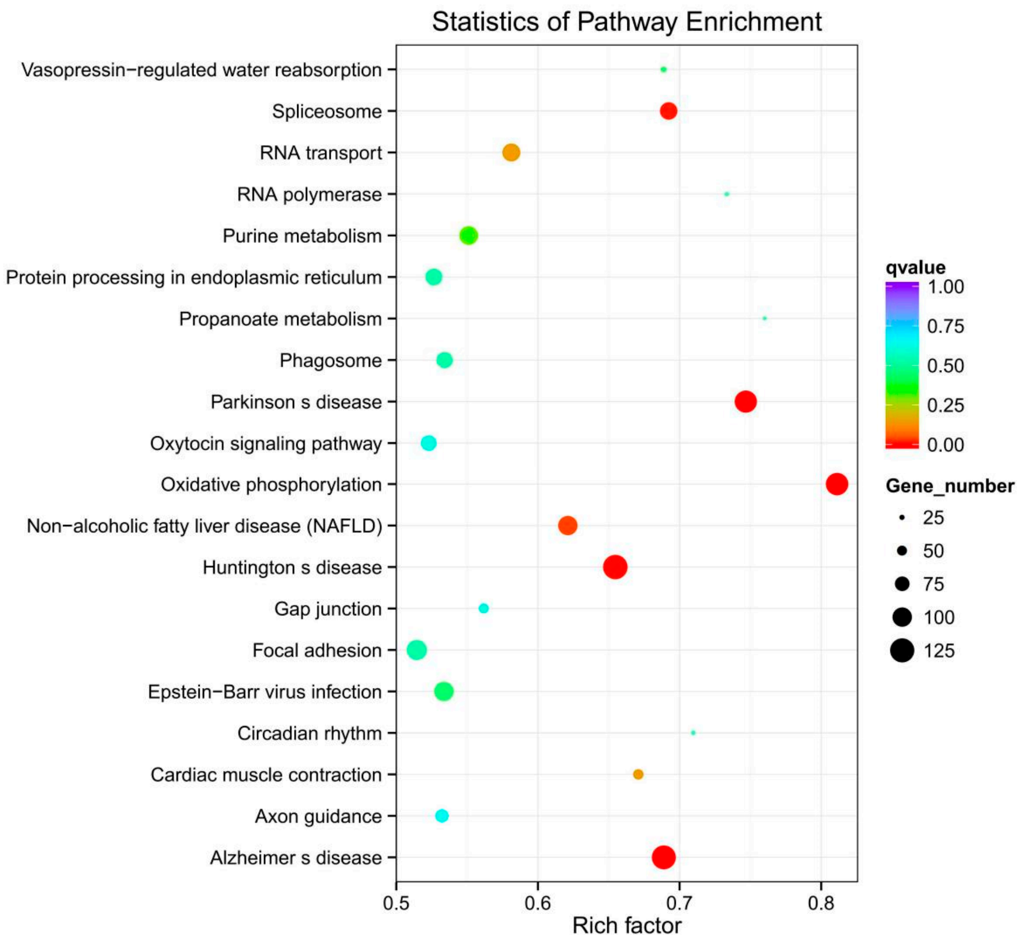

Figure S2.KEGG Pathway Enrichment Map

Note: The size of the circle represents the number of genes enriched in the corresponding pathway, and the larger the circle, the more genes enriched in the pathway; The color represents the enrichment significance, the closer to red, the more remarkable.

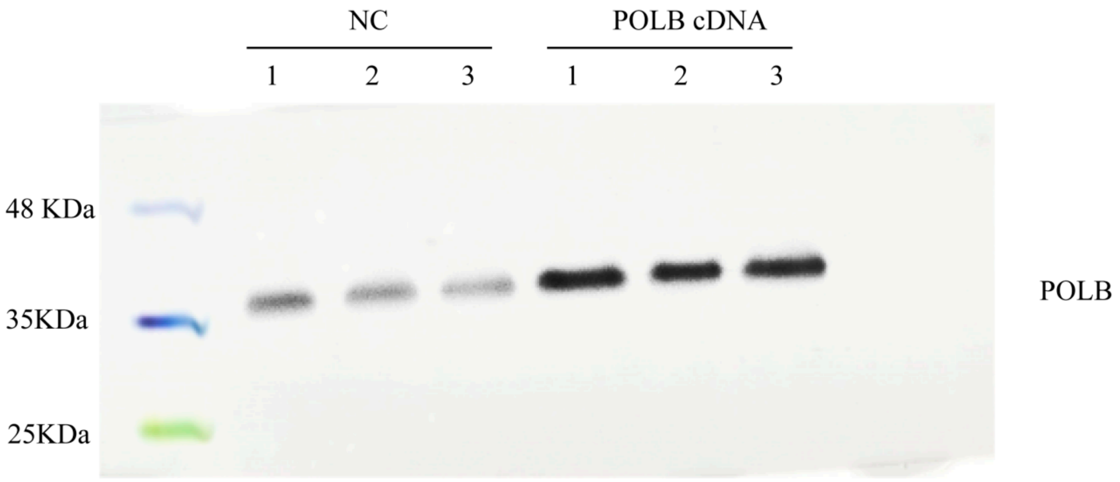

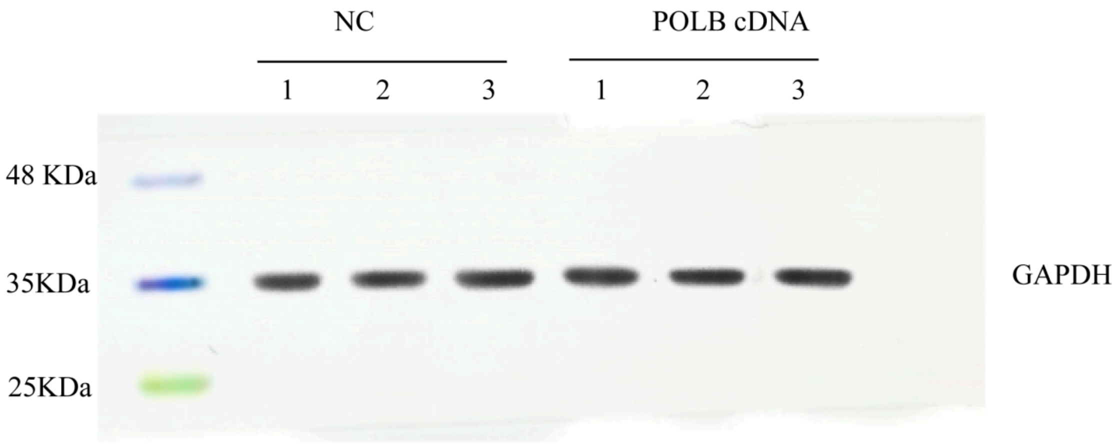

Figure S3. Expression changes of POLB and GAPDH proteins with a negative control (NC) and POLB cDNA construct in bovine primary myocytes.

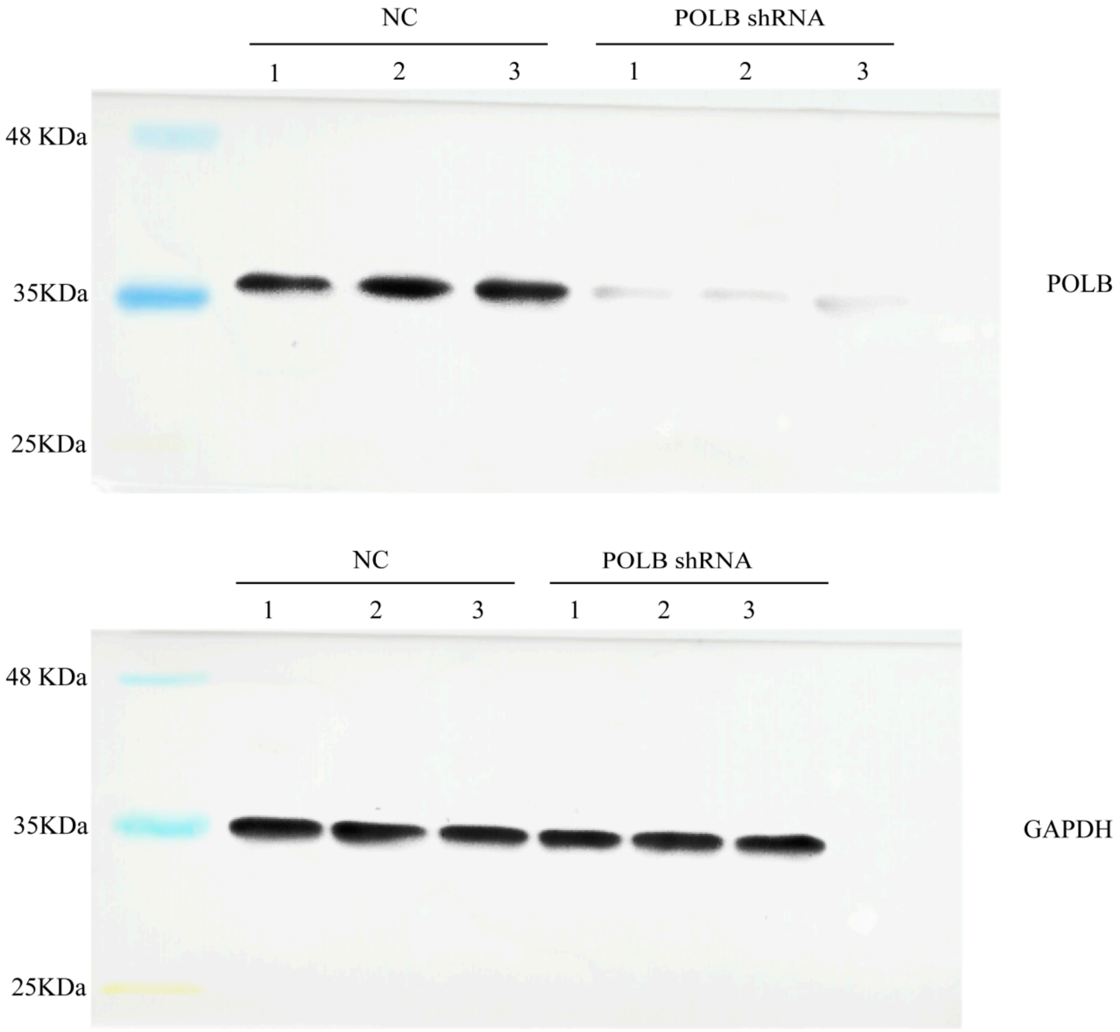

Figure S4. Expression changes of POLB and GAPDH proteins with a negative control (NC) and POLB shRNA construct in bovine primary myocytes.

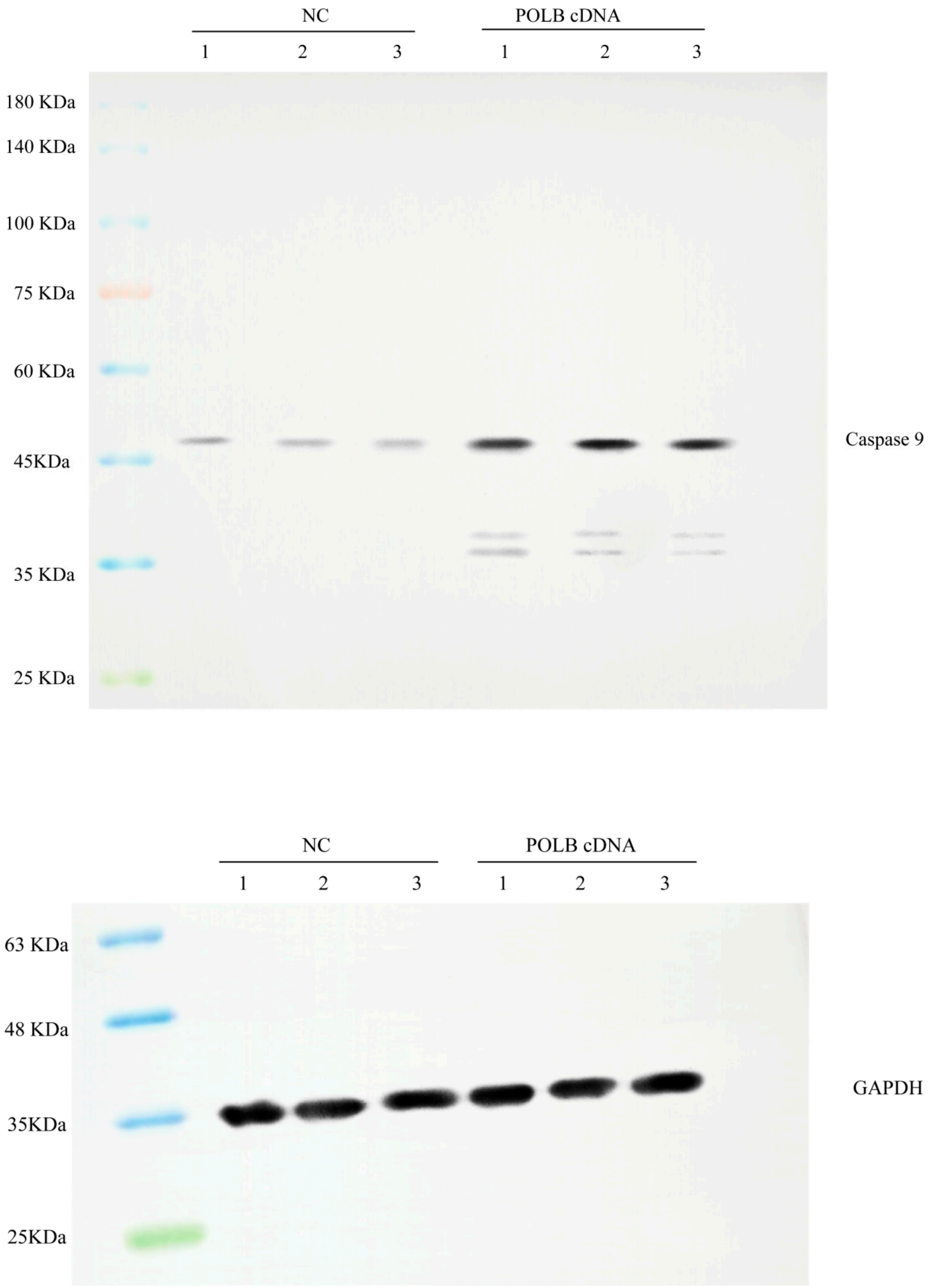

Figure S5. Expression changes of Caspase9 and GAPDH proteins with a negative control (NC) and POLB cDNA construct in bovine primary myocytes.
